# Meta-analysis of the efficacy and safety of sintilimab for treating advanced non-small cell lung cancer

**DOI:** 10.3892/ol.2022.13545

**Published:** 2022-10-12

**Authors:** Jiejie Xie, Xueyan Wu, Jianmei Wu, Fang Huang, Luning Xu

**Affiliations:** Department of Clinical Pharmacy, Sanming First Hospital, Affiliated Hospital of Fujian Medical University, Sanming, Fujian 353000, P.R. China

**Keywords:** sintilimab, non-small cell lung cancer, chemotherapy, meta-analysis

## Abstract

Lung cancer is the leading cause of death from malignant tumors in China, and non-small cell lung cancer (NSCLC) accounts for >80% of all types of lung cancer. Novel immunotherapeutic agents targeting programmed death protein-1 (PD-1) and its ligands [programmed death ligands (PD-L)1 and 2] have emerged as important therapeutic measures and trends in the treatment of advanced NSCLC. Sintilimab (trade name, Daboshu) is a humanized IgG4 monoclonal antibody targeting PD-1 that was developed by Innovent Biologics and Eli Lilly and Company. Studies have shown that sintilimab has the characteristics of high affinity, long-lasting stability and an increased target occupancy rate, and that it is a broad-spectrum drug. The present study uses a meta-analysis to evaluate the safety and efficacy of sintilimab in treating advanced NSCLC. The PubMed, Cochrane Library, Embase, Chinese Biomedical Literature, China National Knowledge Infrastructure, VIP Chinese Science and Technology Journal and Wanfang Medical databases were searched from their establishment until October 2021. Randomized controlled trials of patients with NSCLC who were treated with conventional chemotherapy (chemotherapy) plus sintilimab treatment or sintilimab alone, compared with a chemotherapy group, were included, and a meta-analysis was performed using RevMan 5.3 software. The findings revealed that there was no significant difference between the conventional chemotherapy group and the sintilimab group in terms of the overall incidence of adverse drug reactions (ADR) or grade 3–5 ADRs (risk ratio, 1.04; 95% confidence interval, 0.96-1.14; P=0.33), respectively. Sintilimab coupled with chemotherapy outperformed standard chemotherapy in treating advanced NSCLC. The ADRs did not differ considerably from those of conventional chemotherapy, which will help in the assessment of the clinical efficacy of sintilimab combined with chemotherapy and conventional chemotherapy in treating advanced NSCLC.

## Introduction

Lung cancer is a malignant tumor with the highest morbidity and mortality rates, and the fastest growth rate globally, with non-small cell lung cancer (NSCLC) accounting for 80–85% of cases (1,2). In the early stages of lung cancer, there are no visible signs. Even when there are symptoms, the non-specificity of the clinical presentation affects the speed and accuracy of diagnosis. Nearly 80% of the initial diagnoses are made at the advanced stage (3), excluding the possibility of surgical therapy.

The typical treatment approach for patients with advanced NSCLC who are negative for driving gene mutations is platinum-based combination chemotherapy. The traditional treatment scheme is inefficient and has a high rate of adverse responses (4), decreasing the quality of life and treatment compliance of patients. For advanced NSCLC positive for driver gene mutations, the first-line treatment is targeted therapy, which can effectively prolong the survival of patients and improve their quality of life (5,6). However, at the moment, the positive rate of driving gene mutation in patients with advanced NSCLC is >50% (7,8), and so approximately one-half of the patients are ineligible for targeted treatment.

NSCLC was formerly considered to be a non-immune-related malignancy. Nonetheless, recent 5-year investigations have revealed that negative immune regulation centered on aberrant immunological checkpoints plays an important role in the formation and progression of NSCLC (9–11). With the development of immunology and precision medicine, novel immunotherapeutic agents targeting programmed death protein 1 (PD-1) and its ligands (PD-L1 and PD-L2) have become important measures and trends in the treatment of advanced NSCLC.

Sintilimab (trade name, Daboshu) is a humanized IgG4 monoclonal antibody targeting PD-1 developed by Innovent Biologics and Eli Lilly and Company (12,13). On December 24, 2018, the China Food and Drug Administration formally authorized sintilimab to treat recurrent or refractory classical Hodgkin lymphoma following at least second-line system chemotherapy (12). According to a recent study on sintilimab, the drug is a broad-spectrum medication with strong affinity, long-lasting stability and an enhanced target occupancy rate (14). Sintilimab is now being tested in various stage I, II and III clinical studies in China to treat a range of solid malignancies, including NSCLC and esophageal cancer. To the best of our knowledge, in the domestic and foreign literature to date, there are no meta-analyses on the use of sintilimab for advanced NSCLC. Meta-analyses of PD-1/PD-L1 receptor inhibitors for advanced NSCLC treatment have only included a small number of sintilimab-related studies (15,16). A meta-analysis was performed in in the present study to assess the safety and effectiveness of sintilimab in the treatment of advanced NSCLC, in order to serve as a model for the therapeutic use of sintilimab in advanced NSCLC.

## Materials and methods

### Inclusion and exclusion criteria

#### Inclusion criteria

The inclusion criteria were as follows: i) Study type: Randomized controlled trial (RCT), with the language limited to Chinese or English; ii) Subjects: Patients with NSCLC diagnosed by cytology or pathology; iii) Intervention measures: The trial group received sintilimab alone, or sintilimab combined with chemotherapy, and the control group received general chemotherapy; iv) Evaluation indicators: Overall survival (OS) time, progression-free survival (PFS) time, overall effective rate, objective remission rate, overall adverse drug reaction (ADR) incidence and grade 3–5 ADR incidence (17).

### Exclusion criteria

The exclusion criteria were as follows: i) Literature that does not meet the diagnostic criteria; ii) literature on animal experiments; iii) non-RCT studies; iv) repeatedly published literature; and v) incomplete outcome indicators.

### Document retrieval

Chinese and English databases, including PubMed (https://pubmed.ncbi.nlm.nih.gov/), The Cochrane Library (https://www.cochranelibrary.com/), Embase (www.embase.com/), Chinese Biomedical Literature (http://www.sinomed.ac.cn/index.jsp), China National Knowledge Infrastructure (https://www.cnki.net/), VIP Chinese Science and Technology Journal (http://www.cqvip.com/) and Wanfang Medical (https://www.wanfangdata.com.cn/) databases, were searched. The search time limit was from the establishment of each database until October 2021. The language of publication was limited to Chinese and English. The key words used for the search in both languages were ‘sintilimab’ and ‘non-small cell lung cancer’, with ‘NSCLC’ also used in the English key word search.

### Literature screening and data extraction

Two researchers conducted the literature screening and data extraction independently, and the included information was cross-checked. Differences were resolved by discussion with a third party. The contents of the data extracted mainly included the following: i) General data, such as the study title, author, literature source and publication year; ii) intervention measures and trial implementation in the test group and control group; iii) relevant elements of research type and bias risk assessment; and iv) outcome index and result.

### Document quality evaluation

The selected literature was evaluated according to the evaluation criteria of the Cochrane bias risk assessment tool (18). The evaluation items included whether the random allocation method was correct, whether there was allocation concealment, whether the blind method was implemented, whether the result data were complete, whether there was selective reporting of the research results and whether there were other sources of bias.

### Statistical analysis

RevMan5.3 software (The Cochrane Collaboration) was used for the statistical data analysis. The evaluation indexes OS and PFS used the risk ratio [hazard ratio (HR)] for the statistical quantity of effect analysis; objective remission rate, overall effective rate, overall ADR incidence and drug grade 3–5 ADR incidence were the binary variables. The results used relative risk (RR) as the statistical quantity of effect analysis. The difference was considered statistically significant when the confidence interval (CI) was limited to 95%, with P<0.05. The χ^2^ test was used as the heterogeneity test. When P>0.1 and I^2^<50%, this indicated that there was no heterogeneity among the research results, and the fixed effects model was used for the analysis. When P≤0.1 and I^2^≥50%, this indicated that there was statistical heterogeneity among the research results, and a random effects model was used for the analysis.

## Results

### Basic information of included studies

**Figure 1.**
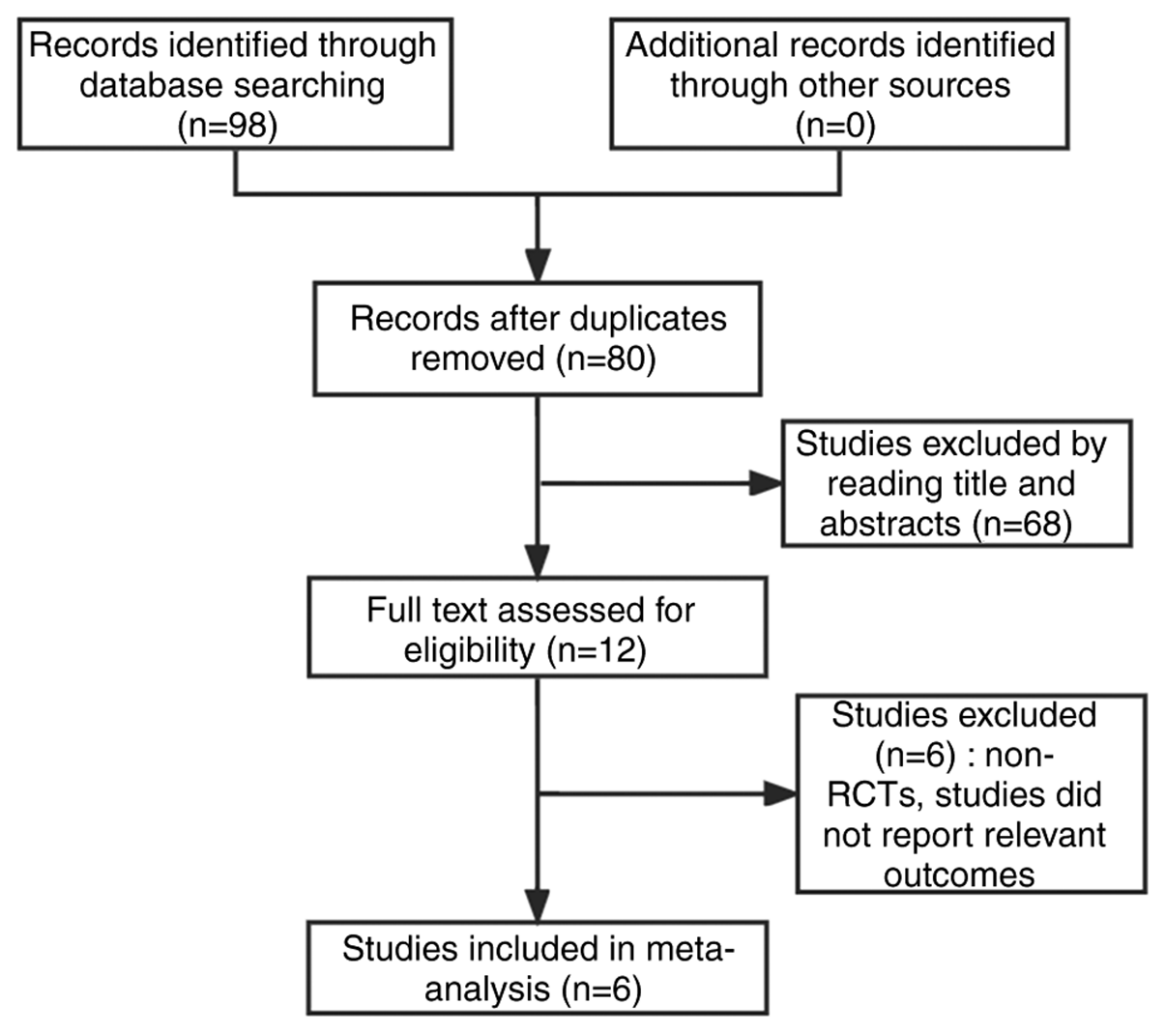
Flow chart of literature screening. RCT, randomized controlled trial.

According to the established literature retrieval strategy, 98 articles were obtained. Following application of the exclusion criteria, 6 articles, including 1,162 patients, were finally included in the meta-analysis. The screening process is shown in Fig. 1, and the essential information from the 6 studies are shown in Table I.

### Document quality evaluation

**Figure 2.**
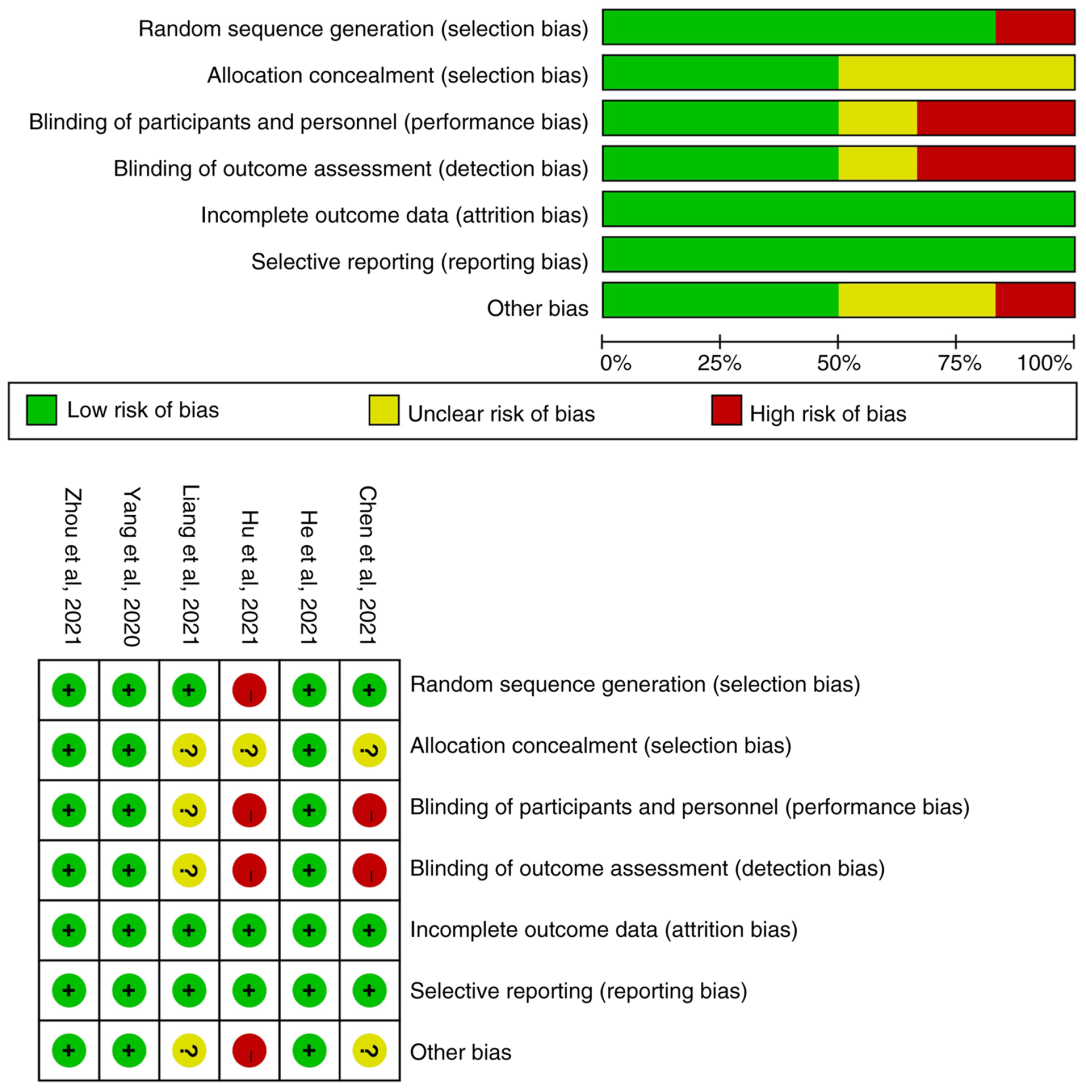
Quality evaluation of incorporated randomized controlled trials. ‘+’ indicates that the risk bias is assessed as low risk, ‘?’ indicates that the risk bias is assessed as ‘uncertain’ and ‘-’ indicates that the risk bias is assessed as high risk.

The Cochrane bias risk assessment tool of RevMan5.3 software was used to evaluate the quality of the 6 RCT studies. It was determined that 3 studies were of good quality and that 3 had a high/unclear risk of bias. The specific literature quality is shown in Fig. 2.

### Meta-analysis results

#### OS time

**Figure 3.**
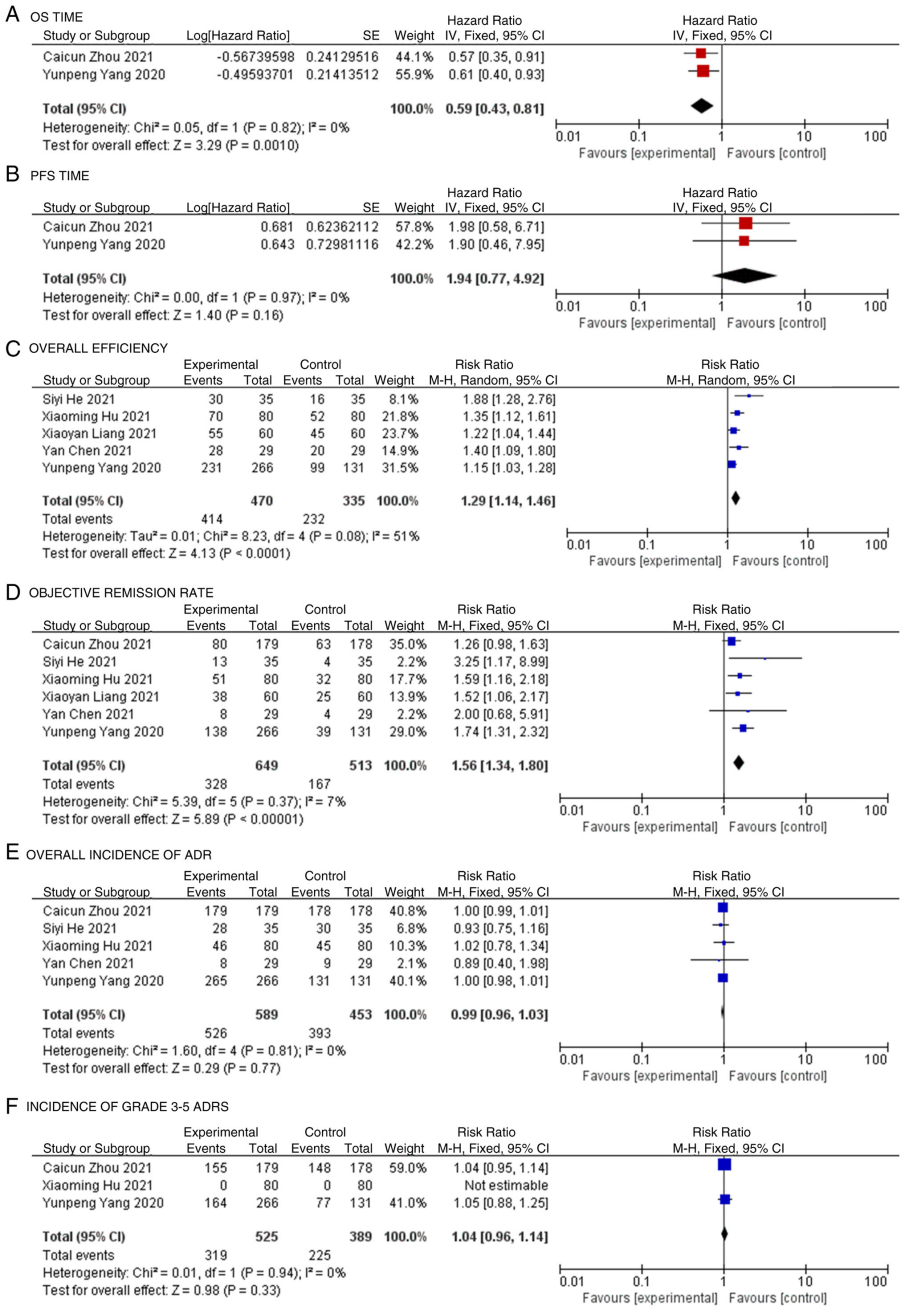
Forest map. Forest map of the comparisons of (A) overall survival, (B) progession-free survival, (C) overall effective rate, (D) objective remission rates, (E) the total incidence of ADRs and (F) the incidence of grade 3–5 ADRs between the experimental group and the control group. ADR, adverse drug reaction; CI, confidence interval.

Among the 6 included studies, only 2 reported the HR value and 95% CI for OS, and these 2 were incorporated into the meta-analysis (19,20). The research results showed no significant difference in heterogeneity (P=0.82; I^2^=0%; Fig. 3A), so the fixed effects model was used for the statistical analysis. The meta-analysis showed that the test group exhibited significantly higher OS times compared with the control group in the patients with NSCLC (HR, 0.59; 95% CI, 0.43-0.81; P=0.0010; Fig. 3A).

### PFS time

Only 2 studies reported the HR value and 95% CI for PFS among the 6 articles, and these 2 papers were incorporated into the meta-analysis (19,20). There was no significant heterogeneity between the results of each study (P=0.97; I^2^=0%), so the fixed effects model was used for the statistical analysis. The meta-analysis showed that the test group exhibited higher PFS times compared with the control group in the patients with NSCLC; however, the difference was not statistically significant (HR, 1.94; 95% CI, 0.77-4.92; P=0.16; Fig. 3B).

### Overall efficiency

Among the 6 included studies, 5 reported the overall effective rate, and these 5 studies were combined for the meta-analysis (19,21–23). The heterogeneity difference among the research results was statistically significant (P=0.08; I^2^=51%), so the random effects model was used for the statistical analysis. The meta-analysis showed that the overall effective rate of the test group was significantly higher than that of the control group (RR, 1.29; 95% CI, 1.14-1.46; P<0.0001; Fig. 3C).

### Objective remission rate

The objective remission rate was reported in all 6 studies, and these 6 studies were combined for the meta-analysis (19–24). There was no significant heterogeneity among the research results (P=0.37; I^2^=7%), so the fixed effects model was used for the statistical analysis. The meta-analysis showed that the objective remission rate of the test group was significantly higher than that of the control group (RR, 1.56; 95% CI, 1.34-1.80; P<0.00001; Fig. 3D).

### Overall incidence of ADR

Among the 6 studies, 5 reported the overall incidence of ADRs, and these 5 were included in the meta-analysis (19,20,22–24). The results showed no significant difference in heterogeneity (P=0.81; I^2^=0%), so the fixed effects model was used for the statistical analysis. The meta-analysis showed no significant difference in the overall incidence of ADRs between the test group and the control group (RR, 0.99; 95% CI, 0.96-1.03; P=0.77; Fig. 3E).

### Incidence of grade 3–5 ADRs

Among the 6 studies, 3 reported the incidence of grade 3–5 ADRs. In the 3 studies included in the meta-analysis (19,20,23), there was no statistically significant difference in heterogeneity among the results (P=0.94; I^2^=0%), so the fixed effects model was used for the statistical analysis. The meta-analysis showed no statistically significant difference in grade 3–5 ADR incidence between the experimental and control groups (RR, 1.04; 95% CI, 0.96-1.14; P=0.33; Fig. 3F).

## Discussion

Lung cancer is the leading cause of death from malignant tumors in China, and NSCLC accounts for >80% of cases (1). Although targeted and traditional therapy has brought significant survival benefits to patients with advanced NSCLC, disease progression and drug resistance have become critical issues limiting existing treatment application and the development of new treatments. The discovery and clinical application of immune checkpoint inhibitors has provided significant progress in the treatment of a variety of solid and non-solid malignant tumors. The PD-1/PD-L1 pathway, as the final rate-limiting step of the antitumor immune response, can effectively block the inhibitory regulation of immune checkpoints and strengthen the antitumor immune response, which brings new hope for the treatment of patients with advanced NSCLC (25). The National Comprehensive Cancer Network guidelines (26) and the Chinese Society of Clinical Oncology (27) recommend immunotherapy combined with chemotherapy as the first-line treatment for PD-L1-positive NSCLC. Nivolumab and pembrolizumab, approved for marketing in Japan and the United States, respectively, in 2015, are the earliest PD-1 inhibitors with significant clinical efficacy (28). However, there are also ADRs such as immune-associated pneumonia, enteritis and rash. As the first domestic PD-1 inhibitor on the market, sintilimab is able to effectively block the PD-1/PD-L1 pathway. Compared with nivolumab and pembrolizumab, sintilimab has different binding epitopes and a greater binding affinity for PD-1 (14). The receptor occupancy rate is >95%, and the effect is lasting and stable; sintilimab has a similar antitumor effect and better safety in advanced NSCLC (29). In June 2021, sintilimab was approved by the National Medical Products Administration for the first-line treatment of advanced squamous NSCLC in combination with chemotherapy. Further clinical trials of sintilimab will change the treatment pattern of lung cancer. At present, clinical trials have been performed on tumor types including, but not limited to, second-line squamous NSCLC (NCT03150875; ORIENT-3), first-line squamous NSCLC (NCT03629925; ORIENT-12), first-line non-squamous NSCLC (NCT03607539; ORIENT-11), and locally advanced epidermal growth factor receptor (EGFR)-mutated or metastatic non-squamous NSCLC (NCT03802240; ORIENT-31) previously treated with EGFR-tyrosine kinase inhibitor (30).

According to the results of the present study, sintilimab combined with chemotherapy can significantly improve the overall disease efficiency and objective remission rate of patients with NSCLC, simultaneously prolonging the PFS and OS times of patients. Sintilimab is superior to ordinary chemotherapy in the treatment of NSCLC. OS time is the time from the beginning of randomization to death (for any reason). Improving the OS time of patients with advanced tumors is the main goal of clinical treatment, and it is also the most important gold standard to evaluate the efficacy of certain antineoplastic drugs. PFS time is the time between the randomization of the patient and the progression of tumor (any aspect) or death (for any reason). To a certain extent, PFS can reflect the quality of life of patients with advanced tumors, and it is also a highly valued indicator in clinical treatment. However, since only 2 studies (19,20) extracted the data for PFS and OS time in the present meta-analysis, the long-term efficacy of sintilimab combined with chemotherapy in the treatment of NSCLC was not determined. Regarding safety, the overall incidence of ADRs to sintilimab combined with chemotherapy was similar to that of conventional chemotherapy, with no statistically significant difference. The common adverse reactions included anemia, neutropenia, leukopenia, thrombocytopenia, nausea, vomiting, diarrhea, rash, liver function damage and renal function damage. The incidence of grade 3–5 adverse reactions of sintilimab combined with chemotherapy was slightly higher than that of conventional chemotherapy, but the results of the meta-analysis showed that there was no statistically significant difference, and the types of grade 3 and above adverse reactions of the two groups were similar, mainly including leucopenia, thrombocytopenia, neutropenia, immune-associated pneumonia, hyponatremia and rash (19,20), which is consistent with that reported in the literature (31). In addition, there have been reported adverse reactions associated with immunotherapy during the treatment of sintilimab (19,20,22). PD-1 inhibitors activate immune cells to kill tumor cells, and this non-specific activation of the immune system may lead to immune damage to other organs and tissues. Most immunotherapy-related adverse reactions occurred within 1–6 months after the initiation of treatment (32), with varying periods, and their clinical manifestations were mostly non-specific, atypical and reversible. Among the literature included in the present meta-analysis, 3 studies (19,20,22) reported the occurrence of adverse reactions associated with immunotherapy. The typical adverse reactions associated with immunotherapy included myelosuppression, hypothyroidism, hyperthyroidism, fever, immune-associated pneumonia, diarrhea, skin reaction, immune-related hepatitis and glomerulonephritis. Nevertheless, the overall incidence of drug withdrawal and mortality caused by sintilimab combined with chemotherapy due to ADRs was low, indicating that it is well tolerated and the safety is controllable.

The present study aimed to evaluate the efficacy and safety of sintilimab in the treatment of advanced NSCLC by comprehensively searching the relevant literature, and by combining and analyzing the results according to the statistical methods of evidence-based medicine, so as to provide the evidence-based basis for the clinical treatment of advanced NSCLC with sintilimab. However, this study still has certain limitations: i) Some of the evaluation indicators in the literature with survival as the endpoint was not included as the data could not be converted to an HR value for the meta-analysis; ii) in the included studies, there is specific heterogeneity in the study design, follow-up time and outcome index data, and the quality of some of the included literature was only average; iii) sintilimab has a short time to market, there are few published associated clinical randomized controlled studies, and the number of included cases is also limited.

Compared with ordinary chemotherapy, the efficacy and safety of sintilimab combined with chemotherapy in the treatment of advanced NSCLC is worth affirming, with a particular clinical application value and being worthy of clinical promotion. However, limited by the number and quality of included studies, the aforementioned conclusions still need to be verified by further large-scale and high-quality studies.

## Figures and Tables

**Table I.** Baseline characteristics included in the randomized controlled trials.

					Intervention measures			
						
First author/s, year	Research type	Location	No. of patients	Diagnosis	Test group	Control group	Outcome index	(Refs.)
Yang *et al*, 2020	Polycentric, random, double-blind, phase III	China	397	Non-squamous non-small cell carcinoma, stage IIIB-IV, EGFR and ALK mutation-negative	Sintilimab (200 mg) combined with pemetrexed and platinum chemotherapy	Placebo combined with pemetrexed and platinum chemotherapy	PFS, OS, objective remission rate, ADR incidence rate, incidence of grade 3–5 ADRs	(19)
Zhou *et al*, 2021	Polycentric, random, double-blind, phase III	China	357	Lung squamous cell carcinoma, stage IIIB/IIIC or IV, EGFR, ALK mutation-negative	Sintilimab (200 mg) combined with gemcitabine/platinum chemotherapy	Placebo combined with gemcitabine and platinum chemotherapy	PFS, OS, objective remission rate, ADR incidence rate, incidence of grade 3–5 ADRs	(20)
Liang and Wei, 2021	Single-center, random	China	120	Advanced non-small cell lung cancer	Sintilimab (200 mg) combined with gemcitabine chemotherapy	Gemcitabine chemotherapy	Overall effective rate, objective remission rate	(21)
He *et al*, 2021	Single-center, random	China	70	Non-small cell lung cancer stage IV, EGFR, ALK mutation-negative	Sintilimab (200 mg) combined with albumin paclitaxel chemotherapy	Albumin-bound chemotherapy	Overall effective rate, objective remission rate, ADR incidence rate	(22)
Hu *et al*, 2021	Single-center, random	China	160	Lung squamous cell carcinoma, NSCLC stage IV	Sintilimab (200 mg) combined with gemcitabine chemotherapy	Gemcitabine chemotherapy	OS, overall effective rate, objective remission rate, ADR incidence rate, incidence of grade 3–5 ADRs	(23)
Chen *et al*, 2021	Polycentric, Random	China	58	Non-small cell lung cancer phase IIIB and IV	Sintilimab (200 mg) combined with anlotinib chemotherapy	Anlotinib chemotherapy	PFS, overall effective rate, objective remission rate, ADR incidence rate	(24)

OS, overall survival; PFS, progression-free survival; ADR, adverse drug reaction.

## Data Availability

The datasets generated during and/or analyzed during the current study are available from the corresponding author on reasonable request.
